# Modeling the relationships between cognitive-linguistic skills and writing in Chinese among elementary grades students

**DOI:** 10.1007/s11145-012-9411-6

**Published:** 2012-09-16

**Authors:** Pui-sze Yeung, Connie Suk-han Ho, David Wai-ock Chan, Kevin Kien-hoa Chung

**Affiliations:** 1 Faculty of Education, The University of Hong Kong, Pokfulam Road, Pokfulam, Hong Kong; 2 Department of Psychology, The University of Hong Kong, Pokfulam Road, Pokfulam, Hong Kong; 3 Department of Educational Psychology, Faculty of Education, The Chinese University of Hong Kong, Shatin, Hong Kong; 4 Department of Special Education and Counselling, The Hong Kong Institute of Education, Tai Po, Hong Kong

**Keywords:** Chinese, Handwriting, Spelling, Ideation, Text writing

## Abstract

The present study is a four-year longitudinal study examining the important predictors of writing of 340 Chinese children in elementary grades. Children’s transcription skills (handwriting skills and spelling), and syntactic skills in grade 1 were significant predictors of text writing in grade 1–4 while ideation in grade 1 only contributed to text writing in grade 2. Stroke order knowledge was shown as an important handwriting skill in Chinese reflecting the characteristics of the Chinese orthography. A model of Chinese writing in early elementary grades was proposed. In the model, orthographic knowledge, morphological awareness and handwriting skills are proposed to contribute to spelling which is correlated with text writing. Handwriting skills, ideation, and syntactic skills were found to contribute to text writing. Path analysis results suggest that the longitudinal relationship between spelling and text writing is bidirectional.

## Introduction

The acquisition of literacy can be defined as acquiring the ability to both comprehend and produce written text (Juel, Griffith, & Gough, 1986). Over the years, more research efforts have been focused on reading and oral language than writing. In a recent review of the literature on writing, Miller and McCardle (2011) pointed out the need for more focused research on the early precursors to writing. While the gap is beginning to close in research on alphabetic languages, research on writing in Chinese has a long way to catch up. Most research on Chinese literacy development focused on reading and character writing (e.g., Chan, Ho, Tsang, Lee, & Chung, 2006; Shu, McBride-Chang, Wu, & Liu, 2006; Tong, McBride-Chang, Shu, & Wong, 2009; Yeung et al., 2011b). There are relatively few studies on text writing and still less on investigating writing development at both word and text levels.

### Writing in alphabetic languages

#### Models of writing

The model proposed by Hayes and Flowers (1980), based on think-aloud protocols of adults, has been one of the most classic models of the cognitive processes in written composition. This model specified the writing process as the interaction among three major processes: planning, translating, and reviewing. An equally influential model is the knowledge-telling model proposed by Bereiter and Scardamalia (1985, 1987) which conceptualizes how beginning writers solve the problems of text writing. According to the model, beginning writers, without the inputs from conversational partners, have to make use of other cues (such as topic, discourse schema and text already produced) for retrieving an item of content which is then subjected to tests of appropriateness. Oral language production and text writing share a lot of similarities among elementary school students as both were found to involve little planning or goal-setting. Around the same time, a simple view of writing, as an analogy of the simple view of reading, was proposed (Juel et al., 1986). It suggests that spelling and ideation (i.e., the generation and organization of ideas) are the primary influences on writing just like word recognition and listening comprehension skills are the proximal causes of reading comprehension. The significance of transcription and text generation in beginning writing was advocated in the “simple view of writing” proposed by Berninger and colleagues (Berninger, 2000; Berninger et al., 1997, 2002b; Berninger & Graham, 1998). Translation was thought to develop earlier than planning and reviewing among developing writers (Berninger & Swanson, 1994). To better capture the uniqueness of writing development among children, the translating component in Hayes and Flower’s (1980) model was further elaborated and was proposed to involve two core processes: transcription and text generation (Berninger et al., 1992; Berninger & Swanson, 1994). Transcription skills, including handwriting and spelling, are the tools for transcribing oral language into visible language (Berninger, 2000). Text generation involves the translation of ideas into oral language representation (Berninger et al., 1992). In a graphic model of the “simple view of writing”, writing is represented by a triangle with transcription and self-regulation executive functions occupying the vertices of the base to support text generation at the apex within a working memory environment (Berninger & Amtmann, 2003). That is, transcription and self-regulation executive functions work together to enable the goal of text generation in working memory (Berninger et al., 2002b). The importance of transcription skills and oral language skills in children’s writing development is highlighted in the model.

What other skills may also be important to text writing development? In the “triangle model extended” (Bishop & Snowling, 2004), skills that are related to the context, such as syntactic skills and discourse skills, are important in language processing at the sentence or paragraph level. Text writing requires more concise syntactic style and more complex sentence and discourse structures than oral narration (Roth, 2000). It is logical to expect that syntactic skills do play an important role in text writing. Numerous empirical studies have showed the close relationship between syntactic skills and reading comprehension (e.g., Bentin, Deutsch, & Liberman, 1990; Demont & Gombert, 1996; Gaux & Gombert, 1999; Mokhtari & Thompson, 2006) but less is known about syntactic skills and text writing. Studies on children with learning disabilities (LD) showed that they use fewer complex sentence structures and make more syntactic errors (Anderson, 1982; Morris & Crump, 1982) and have difficulty in using cohesive devices to connect sentences (Hidi & Hildyard, 1983). Yet, the significance of syntactic skills in text writing seems to be a neglected area in studies among normally achieving children (e.g., Babayiğit & Stainthorp, 2010, 2011; Berninger & Swanson, 1994; Bourdin & Fayol, 1994; Juel et al., 1986). Given the limited amount of research on the relationships between syntactic skills and writing, the following section mainly reviews the research findings involving transcription skills and oral language skills.

#### Skills important to text writing

##### Transcription skills

According to Berninger and colleagues (Abbott & Berninger, 1993; Abbott, Berninger, & Fayol, 2010; Berninger, 2000; Berninger, Abbott, Abbott, Graham, & Richards, 2002; Graham, Berninger, Abbott, Abbott, & Whitaker, 1997), transcription skills include subword letter production (handwriting) and word spelling. In the theoretical model of the developmental constraints on writing acquisition by Berninger, Mizokawa, and Bragg (1991), lower-order neurodevelopmental skills, such as rapid, automatic production of alphabet letters (orthographic-motor integration), may constrain children’s ability to transcribe ideas into written language at the first level of constraint when literacy instruction begins (grades 1–3). After children can acquire automaticity in producing written words, higher-level linguistic processes at the level of the word, the sentence, or discourse structure are thought to constrain the composition process at the second level of constraint among intermediate grade children (grades 4–6). It is thus expected that transcription skills are likely to influence text writing only in early elementary grades. However, a number of studies on English text writing showed that transcription skills are robust cognitive processes in writing among both children (Berninger & Swanson, 1994; Bourdin & Fayol, 1994) and adults (Bourdin & Fayol, 2002; Hayes & Chenoweth, 2006). Both handwriting and spelling are related to composition length and quality (Graham et al., 1997). Studies showed that handwriting training (Berninger et al., 1997) and spelling training (Berninger et al., 1998) transfers to improved compositional fluency. There were at least two main reasons behind the close link between transcription skills and writing performance. First, as children’s handwriting and spelling becomes recognizable by others, they become more motivated to communicate using written language leading to improve written expression (Berninger et al., 2002b). Second, the more automatic the low-level transcription skills are, the more the working-memory resources are available for the higher level constructive aspects of composing (Berninger et al., 1992; McCutchen, 1988, 1995, 1996). On the contrary, simultaneously attending to the mechanical requirements for producing text and planning the next unit of text may interfere with the planning processing and negatively affected the complexity and coherence of content integration.

Findings from studies on consistent orthographies suggest that the importance of transcription skills in text writing may be affected by the transparency of the writing systems. For example, the study by Babayiğit and Stainthorp (2010) among Turkish-speaking children in grade 1 to grade 2 showed that working memory and vocabulary were significant longitudinal predictors of writing quality, but not handwriting or spelling. Similar patterns of results were replicated in another longitudinal study among grade 2 to grade 5 Turkish children (Babayiğit & Stainthorp, 2011). The fact that transcription skills did not constrain text generation in Turkish was claimed to be related to the simplicity of the Turkish spelling system and the emphasis of handwriting skills training in the Turkish curricula (Babayiğit & Stainthorp, 2011). Similarly, the study by Mäki, Voeten, Vauras, and Poskiparta (2001) on Finnish, another transparent writing system, also showed nonsignificant relationships between mechanics of writing (assessed by spelling tests) and writing quality. Spelling only predicted later writing quality between grades 1 and 2 but not by grades 2 and 3. Findings from orthographies in the other extreme, i.e., highly opaque orthography, such as Chinese, would provide important evidence to test the hypothesis that the role of transcription skills in text writing development was affected by the transparency of the writing systems.

##### Oral language skills

Ideation and text generation in the simple view of writing (Juel et al., 1986; Berninger et al., 2002b) highlighted the importance of oral language skills in text generation. Oral language skills were powerful predictors of writing quality (Baker, Gersten, & Graham, 2003; Bishop & Clarkson, 2003; Olinghouse & Leaird, 2009). Vocabulary knowledge is one of the most widely examined oral language skills in text writing development (Babayiğit & Stainthorp, 2010, 2011; Olinghouse & Leaird, 2009). The studies by Babayiğit and Stainthorp (2010, 2011) showed that vocabulary knowledge is the most powerful and reliable longitudinal predictor of writing quality. It was suggested that vocabulary knowledge facilitates writing through its effect on the translation of ideas into grammatical sentences which in turn free up more processing resources for higher level writing processes (e.g., revision) (Babayiğit & Stainthorp, 2011). Instead of vocabulary, the classic study by Juel et al. (1986) measures children’s ability to orally present a story about the picture and use the same marking criteria as the writing task. Since this task assesses the translation of ideas in a more direct way, it is expected to be highly predictive of writing and the results supported this hypothesis. The role of such ideation skill in text writing was also examined in the present study.

### Writing in Chinese

#### Characteristics of the Chinese writing system

Since the reader may not be familiar with the Chinese language, we will first briefly describe the main characteristics of the Chinese orthography. The basic graphic unit in Chinese is a character. In most cases, each character represents both a syllable and a morpheme (Hoosain, 1991; Shu et al., 2006). About 4,600–4,900 Chinese characters are commonly used in Hong Kong where the data from this study were gathered (Cheung & Bauer, 2002; Lee, 2000). More than 1,300 new words are encountered in grade one, which imply a large learning load since the beginning of elementary school in Hong Kong (Chung & Leung, 2008). Chinese characters are made up of different strokes, the basic graphic elements of Chinese handwriting. There are eight basic types of strokes (Law, Ki, Chung, Ko, & Lam, 1998). Strokes are combined to form stroke patterns. The role of strokes in processing Chinese characters has been validated by different experimental studies (e.g., Chen & Kao, 2002; Peng & Wang, 1997; Zhang & Feng, 1992; Zhang, Wang, Zhang, & Zhang, 2002). There are standard rules guiding the order for writing the strokes of a character. Memory of these character stroke orders is important for character spelling in Chinese (Present Authors, 2010). Similar to the Turkish curricular (Babayiğit & Stainthorp, 2011), handwriting skills training is heavily emphasized in the Chinese classroom. Drill-and-practice and learning through practiced word writing have been the predominant approaches to learning to read and write Chinese characters starting from the kindergarten grades (Lin et al., 2009; Wu, Li, & Anderson, 1999)

In view of the differences between the Chinese and alphabetic writing systems in the way they represent sound and meaning, cognitive-linguistic skills that are important to learning to write Chinese are expected to be different from those found in alphabetic writing systems. While a substantial amount of studies examining the cognitive-linguistic skills important to Chinese reading and word spelling has accumulated over the past decade, little is known about the developmental skills beginning writers bring to the task of text writing in Chinese.

Just like the model in Juel et al.’s (1986) study, a comprehensive model on writing examines writing at word level and text level and predictors of both spelling and text writing are to be included. Though there is a lack of research on Chinese text writing, there have been quite a number of studies examining the significant predictors of spelling (i.e., word writing to dictation). In the following, we will provide a summary of the literature on skills that are important to Chinese spelling and other cognitive-linguistic skills (handwriting skills, oral language skills and syntactic skills) that might contribute to Chinese text writing based on the models of writing in alphabetic languages.

#### Skills important to Chinese spelling

Recent research shows that orthographic skills and morphological awareness are important contributors to Chinese reading and spelling development (e.g., Tong et al., 2009; Yeung et al., 2011b; Yeung et al., in press). While the regularities of letter combination are important to children learning to write in alphabetic languages, children learning to write Chinese need to be sensitive to the regularities of character structure and the radicals (Yeung et al., 2011b). Knowledge of character structure was repeatedly found to predict character spelling among Chinese children (Chan et al., 2006; Ho, Chan, Chung, Lee, & Tsang, 2007; McBride-Chang & Ho, 2005; Tong et al., 2009). Besides, radical knowledge (both positional and functional) was closely correlated with Chinese spelling (Ho, Ng, & Ng, 2003; Yeung et al., 2011b; Yeung et al., in press). In a 3-year longitudinal study, children’s grade 1 performance in a pseudo-character meaning judgment task assessing children’s overall knowledge of the position, function, and semantic category of semantic radicals significantly predicted grade 3 Chinese word spelling performance after controlling for autoregressor effects and the contribution from measures of rapid naming, phonological awareness and morphological awareness (Yeung et al., in press). The same measure of orthographic skills also significantly contributed to word reading and spelling in the context of rapid naming, phonological awareness and morphological awareness among first graders (Yeung et al., 2011b).

Morphological awareness is related to spelling among English-speaking children (Carlisle, 1995; Roman, Kirby, Parrila, Wade-Woolley, & Deacon, 2009) and Chinese children (McBride-Chang, Shu, Zhou, Wat, & Wagner, 2003; McBride-Chang et al., 2005; Shu et al., 2006; Tong et al., 2009; Yeung et al., 2011b). Yet, the conceptualization of morphological awareness differs between the two languages. The ability to distinguish among meanings of homophones (i.e., homophone awareness) and morpheme construction skills were the two most important types of morphological awareness that contributed to Chinese word reading, spelling and reading comprehension (McBride-Chang et al., 2003, 2005; Shu et al., 2006). The contribution of homophone awareness to Chinese word dictation (i.e., spelling) was found to be stronger than that of morpheme construction skills in a 3-year longitudinal study among Chinese elementary students (Yeung et al., in press). The same measure of homophone awareness was therefore adopted as a measure of morphological awareness in the present study.

#### Skills important to Chinese text writing

##### Handwriting skills

There was a lack of direct evidence on the role of handwriting skills in writing development among Chinese children. Still, findings in a study by Tan, Spinks, Eden, Perfetti, and Siok (2005) hinted that handwriting skills are likely to be important to Chinese writing development. Their results suggested that orthographic awareness was strongly related to reading ability among beginning and intermediate readers while motoric programs that subserve writing, as measured by a picture drawing task, explained significant amount of variance of reading among intermediate readers. The motor activity in Chinese character writing facilitates the formation of long-term motor memory and consolidation of the lexical representation of Chinese characters by pairing the hand movement patterns and language stimuli. These echoed the findings that motor skills and orthographic coding contributed to handwriting among English writers (Abbott & Berninger, 1993).

A distinctive feature of Chinese writing is stroke order. Writing Chinese characters according to the correct stroke orders is one important basic handwriting skill emphasized in classroom teaching. In traditional Chinese classrooms, teachers teach writing by demonstrating writing each character stroke-by-stroke on the blackboard and tracing the individual strokes with hand movements in the air. Students learn by copying the character by following the stroke order presented by the teacher and imitating teacher’s movements of tracing individual strokes in the air (Packard et al., 2006). Unlike spelling in English, there is no nameable component (e.g., alphabets) in Chinese. The psychomotor memory of the stroke order of words is essential for writing a Chinese character from memory. In general, there are six major rules of stroke order in Chinese writing (Law et al., 1998). Results from the priming study by Flores d’Arcais (1994) suggest that the temporal order of strokes in Chinese characters is likely to be related to the processing of lexical representation. In the study by the Present Authors (2010), they examined children’s ability to produce correct stroke order by asking them to write the next stroke in an incomplete Chinese character with the visual form of the complete Chinese character given next to the stimuli. Performance in this task significantly predicted spelling in grade 1 and 2 students even after controlling for the contribution from rapid naming, phonological awareness, morphological skills, and orthographic knowledge. In order to investigate the contribution of low-level transcription skills to text writing in Chinese, the present study not only examined the contribution of this skill in producing correct stroke order to word spelling but also that to text writing.

##### Oral language skills

The importance of oral language skills in literacy acquisition in alphabetic languages is unquestionable (e.g., Gough & Tunmer, 1986; Hoover & Gough, 1990). In Chinese, the findings on the importance of oral language skills in literacy acquisition were mixed. In the cross cultural study by McBride-Chang et al. (2005), vocabulary knowledge, an oral language skills measure, contributed unique variance to Chinese word recognition after controlling for morphological awareness and phonological awareness among second graders in Hong Kong but not those in Beijing. In the study by Shu et al. (2006), vocabulary knowledge significantly predicted reading but not spelling after controlling for morphological awareness, phonological awareness and rapid naming among fifth and sixth graders in Beijing. Recent research findings among elementary grade children in Hong Kong suggest that vocabulary and oral discourse skills are not significant predictors of Chinese word reading, word spelling or reading comprehension (e.g., Yeung, Ho, Chan, Chung, & Wong, 2011; Yeung et al., 2011b). The role of oral language skills in Chinese text writing was rarely examined in previous studies. Modeling after the ideation task used in the classic study by Juel et al. (1986), an oral sentence construction task was developed in the present study to assess children’s ability to generate ideas. Similar to their design, the marking criteria for the oral sentence construction task was similar to that for the text writing task.

##### Syntactic skills

Recent studies showed that syntactic skills uniquely contribute to Chinese text level literacy, sentence and passage reading comprehension, among early elementary grade children (Chik et al., 2011; Yeung et al., 2011b). In the study by Yeung et al. (2011b), syntactic skills accounted for significant amount of unique variance in Chinese reading comprehension at both sentence and passage levels after controlling for the effects of word reading and other cognitive-linguistic skills (rapid naming, phonological awareness, orthographic skills, and morphological awareness) among grade one students in Hong Kong. Similarly, Chik et al. (2011) showed that syntactic skills were a significant longitudinal predictor of Chinese text-level reading comprehension among first and second graders. However, there has been little empirical evidence on the importance of syntactic skills to text writing. In the studies by Chik et al. (2011) and Yeung et al.’s (2011b), Chinese syntactic skills were operationalized as word order knowledge. Chinese lacks grammatical morphology rendering word order a most important syntactic device in Chinese (Chang, 1992; Chao, 1968).

### Aims of the present study

The present study aims to examine the interrelationships among the cognitive-linguistic skills that are important to Chinese children’s writing development at both word and text levels. Three types of cognitive-linguistic skills thought to have more direct contribution to text writing investigated in the study were transcription skills, ideation and syntactic skills. The former two originated from the simple view of writing (Berninger, 2000; Berninger & Graham, 1998; Juel et al., 1986) while the expected significance of syntactic skills was based on the “triangle model extended” (Bishop & Snowling, 2004). The transcription skill in the present study was operationalized as the accuracy to produce strokes according to correct stroke order. As for ideation, we assessed children’s ability to construct sentences orally based on given themes and the marking criteria were similar to that of text writing. It is hoped that this task can assess the translation of ideas in a more direct way than the oral vocabulary knowledge measure used in most previous studies. Syntactic skills were assessed by a word order knowledge task (Chik et al., 2011) that was found to predict Chinese text reading. In addition to these measures, measures of orthographic awareness and morphological awareness, significant predictors of writing at word level (i.e., word spelling) in Chinese (Chan et al., 2006; Tong et al., 2009; Yeung et al., 2011b), were included for conceptualizing Chinese writing development at both word and text levels.

Based on the model of the developmental constraints on writing acquisition (Berninger et al., 1991), it was hypothesized that syntactic skills, a higher level linguistic skills, were only influential in higher grades but not among students in early grades. In view of the complexity of the Chinese writing system, transcription skills were expected to play an important role in text writing even in more advanced grades as it takes longer time for Chinese writers to attain automaticity in the mechanics of writing. In previous studies on text writing development (e.g., Babayiğit & Stainthorp, 2011; Juel et al., 1986; Mäki et al., 2001), spelling is conceptualized as a longitudinal predictor of text writing but not vice versa. In the present study, we tested the hypothesis about the bidirectional influence between spelling and text writing across time by postulating two different models. In one model, the strength of the influence between spelling and subsequent text writing and that between text writing and subsequent spelling was assumed to be equal while the strength of the relevant paths are free to vary in another model.

## Method

### Participants

Participants of this study were 340 Chinese students whose mother tongue was Cantonese. There were 173 girls and 167 boys recruited from three representative primary schools in Hong Kong with normal intelligence (mean IQ = 110). All three schools used Cantonese as the medium of instruction for Chinese language lessons. Children in Hong Kong encountered about 1,300 new Chinese characters in grade one (36 % of all the new Chinese characters they are to learn in elementary grades) and around 500–600 new characters each year in grades 2–4 (Chung & Leung, 2008). There is no phonetic system, like *pinyin* in the mainland China, to assist Chinese character learning in Hong Kong and children mostly learn to read Chinese characters with a “look and say” method. The children were tested on general reasoning ability, orthographic knowledge, morphological awareness, syntactic skills, ideation, handwriting skills, word spelling and text writing when they were in grade 1 (mean age = 7.08 years) (Time 1), and on word spelling and text writing when they were in grade 2 (mean age = 8.04 years) (Time 2) and grade 4 (mean age = 9.94 years) (Time 3).

### Measures

#### General reasoning ability

To control for the impact of students’ general reasoning ability on their writing performance, they were administered the Raven’s Standard Progressive Matrices. This is a standardized test for measuring general reasoning ability, including five sets of 12 items each. Each item consisted of a target visual matrix with a missing piece. Children were required to pick, from six to eight alternatives, the best part to complete the target matrix. The short form of the test, made up of the first three sets of the full form, was administered to participants of less than 8.5 years old in the present study. Scoring procedures were based on the local norm established by the Education Department of The Hong Kong Government in 1986.

#### Orthographic knowledge

Children’s orthographic knowledge was assessed by the pseudo-character meaning judgment task used in Yeung et al.’s (2011b) study. The task was modeled after a similar task developed in Ho et al.’s (2003) study to measure the children’s overall awareness of positions, functions, and semantic categories of different semantic radicals in written Chinese characters. As mentioned above, orthographic knowledge measured by this task was found to have significant unique contribution to Chinese word reading and spelling on top of age, IQ, and oral vocabulary (Yeung et al., 2011b). In each testing item, a pseudo-character was placed next to four picture choices (see a sample item in the “Appendix”). Each pseudo-character (e.g.,  ) was composed of a semantic radical (e.g., 衤 which carries the meaning of “clothing”) and a phonetic radical (e.g., 太 /taai3/) in their legal positions but the combination was not a real Chinese character. Both lexical semantic radicals (e.g., 皿) and nonlexical semantic radicals (e.g., 疒) were used to construct pseudo-characters. All pseudo-characters were checked against *Kangxi Dictionary* (Zhang, 1979) to ensure that they were not real characters. All the semantic radicals appeared in the task were found in words familiar to the children (Leung & Lee, 2002) and most of them were introduced explicitly in classroom teaching in Grades 1–2. The children were asked to circle the picture that might be semantically related to the meaning of the pseudo-character. The instruction was as follows:

你睇下你本簿上面呢個得意字, 然後你估吓佢嘅意思同隔離呢四幅圖畫中邊一幅係有關係? 試吓圈低你覺得有關嘅圖畫。

[Take a look at the interesting character on the answer book and guess which of the four pictures by its side is associated to its meaning. Circle the associated picture.]

Two practice items were presented before the 16 testing items. One mark was given for the correct answer to each item. Cronbach’s alpha coefficient for this task was 0.67.

#### Morphological awareness

Similar to the study by Yeung et al. (2011b), a homophone awareness task adapted from the morpheme identification task in the study by McBride-Chang et al. (2003) was used to assess children’s morphological awareness in terms of their ability to differentiate morphemes from homophones. In each item, children were presented with three two-syllable Chinese words orally in Cantonese without the use of print, and one of the characters in each of the three words share the same pronunciation (i.e., homophones). Out of the three homophones in each trial, two of them share the same morpheme. The children were asked to identify the two words containing the same morpheme by circling the numbers (1, 2, or 3) assigned to the words according to the presentation order. In the practice trial, children were presented the words /naam4 tsi3/ (男廁 male washroom), /naam4 dzai2/ (男仔 young boy), and /naam4 gik9/ (南極 South Pole) shared the same syllable /naam4/. The first two words contained the homophones sharing the same morpheme (the meaning of male), whereas the homophone in the last word had a meaning of south. Therefore, children would have to circle the number 1 and 2 for that trial (see a sample item in the “Appendix”). The position of the target syllables in the words and the order of correct answers were counterbalanced across items. Two practice trials were given. There were a total of 15 items in this task and one mark was given for the correct answer to each item. Cronbach’s alpha coefficient for this task was 0.64.

#### Ideation

An oral sentence construction task was developed to assess children’s ability to generate ideas. Children were asked to construct a sentence based on a two-character word in each item. The stimuli words belong to one of the three different classes: noun, verb and adjective. There were two practice and five testing items. In the first practice trial, children were given the word “supermarket” (超級市場). They were encouraged to think about the time, place, persons involved and what happened when constructing their sentence. Then a sample sentence was presented, “On Sunday, mum went to a very crowded supermarket to buy groceries.” In the next practice trial, they were given a phrase “going to a park” (去公園) and were asked to construct a sentence on their own. Responses of the children were recorded and scored according to three aspects: content, sentence structure, and usage of words. The content aspect was scored on a scale of 0–2 ranging from ‘poor—mostly irrelevant information’ to ‘good—relevant and detailed’. The sentence structure was scored on a scale of 0–4 ranging from ‘poor—incomplete/incorrect sentence structure’ to ‘good—appropriate use of connectives and subordinate clauses’. Usage of words was scored on a scale of 0–3 ranging from ‘poor—inappropriate choice of vocabulary’ to ‘good—rich and sophisticated choice of vocabulary’. The maximum score for this task was 45. 30 % of the scripts transcribed were independently marked by two well-trained research assistants and the interrater reliability was 87 %. Cronbach’s alpha coefficient for this task with five items was 0.65.

#### Syntactic skills

A word order knowledge task, like the one used in Chik et al.’s (2011) study, was administered to measure the children’s awareness of basic sentence structure rules in written Chinese (e.g., subject-verb-object, subject-verb-verb, etc.). Word order knowledge was found to correlate strongly with other syntactic skills, e.g., connective usage and knowledge of morphosyntactic structure (Chik et al., 2011). In each trial, children were given a number of sentence fragments in randomized order and were asked to arrange them to form a syntactically correct sentence (See Example 1 below). There were four practice trials. To minimize the possible influence of word reading ability, the words in each trial were read aloud to the children. One point was given to each correctly ordered sentence with three to four segments. For the sentence items with five to six segments, the maximum score was two and one point was given to responses with 3–4 segments arranged syntactically correct out of the five to six segments. The acceptable answers (i.e., syntactically correct sequences) to the items were determined by Chinese Language teachers and an expert in Chinese linguistics. There were 10 items with three to four segments and 4 items with five to six segments. The maximum scores for the task were 18. Cronbach’s alpha coefficient for this task was 0.74.

##### *Example 1*

(1) 弟弟 Younger brother / (2) 足球 football / (3) 正在玩 is playing (1) (3) (2)

#### Handwriting skills

To measure the handwriting skills of the participants, a stroke order task used in the study by Present Authors (2010) was adopted. It has been found that stroke sequence knowledge is an important type of orthographic knowledge, apart from radical knowledge, in learning to read and spell Chinese characters among beginning learners (Present Authors, 2010). In each trial, children were presented with a printed Chinese character and an incomplete version of the same printed Chinese character with some strokes missing. Within each pair, one of the characters was printed in black (i.e., complete character) whereas only the first few strokes of the other character within the pair were printed in black (i.e., incomplete character). The remaining strokes of this incomplete character were in grey colour which suggested the unfinished parts of it (see a sample item in the “Appendix”). Participants were asked to fill in the next stroke for the incomplete character. The stroke orders tested were based on the 6 basic stroke rules in Chinese calligraphy including, horizontal before vertical; left before right; top before bottom; outside before inside; diagonals right-to-left before diagonals left-to-right; centre verticals before outside strokes. There were 18 testing items and one mark was given for the correct answer to each item. Cronbach’s alpha coefficient for this task was 0.72.

#### Word spelling (i.e., word dictation)

Chinese spelling ability of the participants was assessed by a word dictation task. Stimuli words were two-character words selected from the two Chinese textbooks commonly used for teaching Chinese among first graders in Hong Kong. In each item, a two-character word was first read aloud by the experimenter. Then a simple sentence containing the two-character word was presented. Afterwards, the two-character word was read aloud again and the children were asked to write down the two-character word only. The number of items (Cronbach’s alpha coefficient) at Time 1, Time 2, and Time 3 were 13 (0.84), 10 (0.82), 12 (0.88) respectively. One mark was given for each correctly written character. The maximum scores for Time 1, Time 2, and Time 3 were 26, 20 and 24 respectively.

#### Text writing

The task was designed to assess participant’s Chinese writing skill. Children were given a theme “開心的生日” and were asked to describe a happy birthday scene. They were allowed to ask experimenters for the writing of any character and incorrect writing of characters was not penalized. The same task was given to the participants at Time 1, Time 2 and Time 3. The answers of the children were scored according to four aspects: content, sentence structure, organization, and usage of words. The content aspect was scored on a scale of 0–3 ranging from ‘poor—mostly irrelevant information’ to ‘very good—relevant and detailed’. The sentence structure was scored on a scale of 0–5 ranging from ‘poor—incomplete/incorrect sentence structure’ to ‘very good—appropriate use of connectives and subordinate clauses’. Organization was scored on a scale of 0–3 ranging from ‘poor—discourse is disorganized’ to ‘very good—discourse is well organized and coherent, with a clear progression of ideas’. Usage of words was scored on a scale of 0–4 ranging from ‘poor—inappropriate choice of vocabulary’ to ‘very good—rich and sophisticated choice of vocabulary’. Twenty percent of the students’ responses were independently marked by two well-trained research assistants and the interrater reliability at Time 1, Time 2, and Time 3 were 0.92, 0.95 and 0.88 respectively.

### Procedures

Most of the tasks in the present study were given to the children in three group sessions (each lasted for 60 min or less) and one individual session (lasted for 15 min or less) at Time 1. The order of tasks in each group or individual session was fixed but the order of the administration of the three group sessions and one individual session was randomized. Tasks that were administered in groups at Time 1 were Raven’s Standard Progressive Matrices, pseudo-character meaning judgment, homophone awareness, word order knowledge, stroke order knowledge, word spelling, and text writing. Oral sentence construction was administered individually at Time 1. Word spelling and text writing were administered in two group sessions (lasted for 30 min each) at Time 2 and Time 3.

## Results

### Descriptive analyses

Table 1 presents the means, standard deviations, and ranges computed for the various tasks in this study. Most of the reliability coefficients are in the medium to high range (0.64–0.95).

**Table 1:** Means, standard deviations and ranges for measures in the present study (*N* = 340)

Variables	Mean	SD	Range	Max
Age (in months) (Time 1)	84.93	4.35	79–116	
Raven’s (Time 1)	111.39	13.83	70–135	
Pseudo-character meaning judgment (Time 1)	10.17	2.98	3–16	16
Homophone awareness (Time 1)	11.08	2.61	1–15	15
Oral sentence construction (Time 1)	21.49	3.93	5–38	45
Word order knowledge (Time 1)	11.36	4.04	1–18	18
Stroke order knowledge (Time 1)	9.98	3.32	1–17	18
Word spelling (Time 1)	10.32	5.00	0–22	26
Word spelling (Time 2)	13.27	4.26	1–20	20
Word spelling (Time 3)	11.45	5.78	0–24	24
Text writing (Time 1)	5.75	3.29	0–15	15
Text writing (Time 2)	6.84	2.73	0–15	15
Text writing (Time 3)	8.49	2.87	0–15	15

Raven’s—Raven’s standard progressive matrices

### Correlation

Table 2 shows the correlations among all the measures in the present study. All correlations among the cognitive-linguistic measures for orthographic knowledge, morphological awareness, ideation, syntactic skills and handwriting skills were significant (*r*s > 0.14, *p*s < 0.05). All measures were significantly correlated with text writing (*r*s > 0.18, *p*s < 0.05).

**Table 2:** Correlations among all variables in this study

	Age (T1)	RA (T1)	OK (T1)	HA (T1)	OSC (T1)	WO (T1)	SO (T1)	WS (T1)	WS (T2)	WS (T3)	TW (T1)	TW (T2)	TW (T3)
Age (T1)	–												
RA (T1)	−0.06	–											
OK (T1)	0.09	0.36	–										
HA (T1)	0.05	0.35	0.47	–									
OSC (T1)	−0.01	0.15	0.17	0.15	–								
WO (T1)	0.03	0.43	0.49	0.45	0.26	–							
SO (T1)	0.05	0.31	0.35	0.25	0.14	0.32	–						
WS (T1)	0.08	0.20	0.31	0.43	0.06	0.46	0.30	–					
WS (T2)	0.15	0.20	0.32	0.24	0.07	0.33	0.21	0.56	–				
WS (T3)	0.05	0.21	0.33	0.34	0.13	0.40	0.33	0.65	0.67	–			
TW (T1)	0.13	0.27	0.26	0.26	0.25	0.42	0.22	0.38	0.28	0.30	–		
TW (T2)	0.04	0.24	0.31	0.25	0.27	0.40	0.28	0.32	0.34	0.41	0.41	–	
TW (T3)	0.15	0.19	0.29	0.23	0.23	0.35	0.18	0.38	0.46	0.48	0.31	0.37	–

*T1* Time 1, *T2* Time 2, *T3* Time 3, *RA* Raven’s standard progressive matrices, *OK* pseudo-character meaning judgment, *HA* homophone awareness, *OSC* oral sentence construction, *WO* word order knowledge, *SO* stroke order knowledge, *WS* word spelling, *TW* text writing. For all variables, *N* = 340, correlations of magnitude 0.12 are significant at *p* < 0.05

### A model of writing in Chinese

**Fig. 1:**
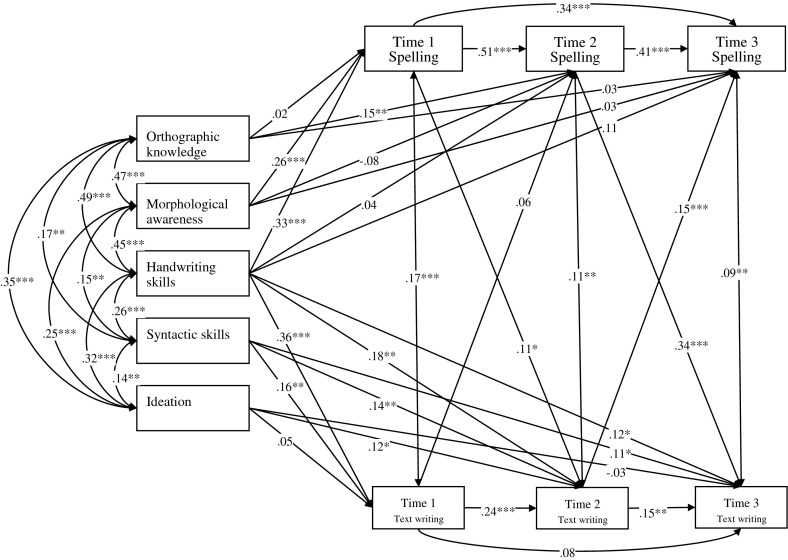
Model 1 of text writing in Chinese. * *p* < 0.05; ** *p* < 0.01; *** *p* < 0.001

**Fig. 2:**
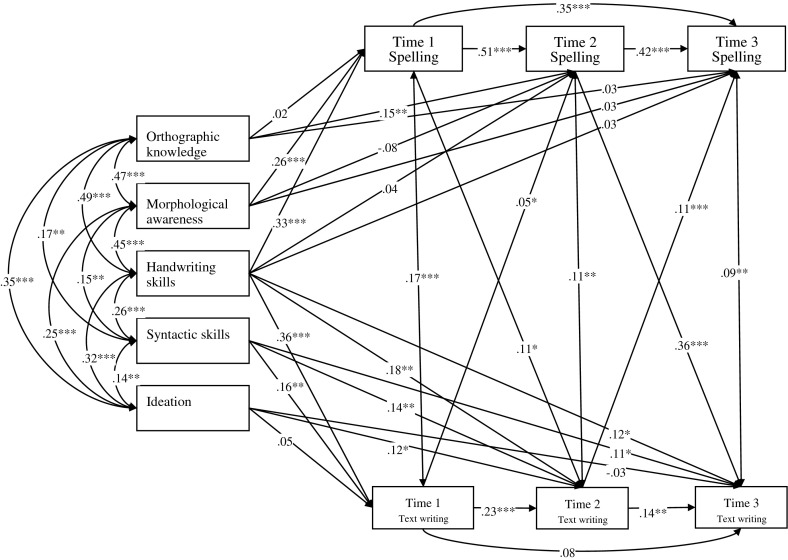
Model 2 of text writing in Chinese. * *p* < 0.05; ** *p* < 0.01; *** *p* < 0.001

In order to model Chinese writing development at word and text levels, the associations among the variables were investigated by running the path analyses using LISREL 8.80, a structural equation modeling program. The first path analyses model (Model 1) served as a baseline model (Fig. 1). Orthographic knowledge, morphological awareness and handwriting skills were postulated to have significant effects on Time 1, Time 2 and Time 3 spelling which in turn correlate with text writing at corresponding time points. Handwriting skills, syntactic skills and ideation were postulated to have direct effects on Time 1, Time 2 and Time 3 text writing. Time 1 and Time 2 spelling were postulated to have direct effects on Time 2 and Time 3 text writing respectively. Time 1 and Time 2 text writing were postulated to have direct effects on Time 2 and Time 3 spelling respectively. The overall fit of Model 1 was good (χ^2^ (13, *N* = 340) = 25.11, *p* = 0.022, Non-Normed Fit Index (NNFI) = 0.98, Comparative Fit Index (CFI) = 0.99 and Root Mean Square Error of Approximation (RMSEA) = 0.053). Results showed that orthographic knowledge had significant direct effect on spelling at Time 2 only while morphological awareness and handwriting skills had significant direct effects on spelling at Time 1 only. Handwriting skills and syntactic skills had significant direct effects on text writing across time while ideation only had significant direct effects on text writing at Time 2. All the postulated paths among spelling at Time 1, Time 2 and Time 3 were significant. As for text writing, the paths from Time 1 to Time 2 and that from Time 2 to Time 3 were significant. Besides, all the postulated paths between spelling and text writing were significant, except for that from Time 1 writing to Time 2 spelling. Model 2 was postulated to test the hypothesis that the relationship between spelling and text writing is bidirectional across time (Fig. 2). The only difference between Model 1 and Model 2 was that the strength of the path from Time 1 spelling to Time 2 text writing was postulated to be equal to that from Time 1 text writing to Time 2 spelling; and the strength of the path from Time 2 spelling to Time 3 text writing was postulated to be equal to that from Time 2 text writing to Time 3 spelling. Significant paths in Model 2 were same as those in Model 1, except that the path from Time 1 text writing to Time 2 spelling was significant in Model 2 but not in Model 1. Model 2 fits the data well (χ^2^ (15, *N* = 340) = 25.78, *p* = 0.040, Non-Normed Fit Index (NNFI) = 0.98, Comparative Fit Index (CFI) = 0.99 and Root Mean Square Error of Approximation (RMSEA) = 0.046). The overall fit of Model 2 was similar than that of Model 1 (Δχ^2^ (2, *N* = 340) = 0.67, *p* > 0.25) but it is a more restricted model than Model 1. Thus, Model 2 is thus the preferred model conceptualizing the interrelationships among the variables.

## Discussion

### A model of Chinese writing development among beginning writers

The present study ventures to postulate a model of Chinese writing development among elementary grade students based on the writing models developed for alphabetic languages (Berninger, 2000; Berninger & Graham, 1998; Juel et al., 1986), the unique characteristics of the Chinese writing system and the previous research on Chinese word writing. Consistent with our hypothesis, transcription skills (both handwriting skills and spelling) play an important role in Chinese text writing development beyond early elementary grades. This might reflect the fact that the relative importance of different cognitive-linguistic skills important to text writing in a specific language is a function of the complexity of its writing system. One unexpected finding was that syntactic skills, instead of ideation, were a strong contributor to text writing development across elementary grades. This unique finding suggests that, other than the orthographic depth of the writing systems, there may be other variable of the language/writing systems to be considered in the formulation of developmental models of text writing. This point will be further elaborated in a later section. Moreover, the path analyses results suggest that the interaction between word spelling and text writing can better be conceptualized as bidirectional, instead of unidirectional from word spelling to text writing.

### Significant predictors of text writing in Chinese

Path analyses results showed that transcription skills and syntactic skills were both significant predictors of Chinese writing among children in elementary grades but the contribution of ideation was relatively limited. One unique finding in the present study was that transcription skills were significant predictors of text writing not only among first and second graders but also fourth graders. This seems to support the suggestion that the significance of transcriptions skills in text writing is not limited to early writers in orthographies that are more opaque and complex, like Chinese. Besides, syntactic skills were found to play an important role in text writing starting in early elementary grades.

### Unique role of stroke order knowledge in Chinese text writing

Handwriting and spelling are correlated but separable skills that may develop independently of each other in some cases (Berninger, 2000). Moreover, there was evidence that handwriting is of particular importance. For example, using structural equation modeling, Graham et al. (1997) showed that while the path from spelling to compositional fluency was significant in the primary grades, the path from handwriting was significant in primary grades and intermediate grades for both compositional fluency and compositional quality. Besides, the contribution of spelling to compositional quality was indirect through its correlation with handwriting. For English, handwriting fluency, but not handwriting quality, was moderately correlated with measure of composing (Biemiller, Regan, & Gang, 1993; Meltzer, Fenton, & Persky, 1985; Parker, Tindal, & Hasbrouck, 1991). Handwriting fluency in English was usually assessed by asking children to print the 26 alphabet letters in order from memory as quickly and accurately as they could within a certain time period (e.g., Berninger et al., 2002b; Graham et al., 1997). While producing alphabet letters quickly is an important transcription skill for writing development in English, results in the present study highlighted the importance of stroke order as a Chinese transcription skills component in Chinese writing development. Though stroke order has been conceptualized as a basic element in Chinese writing system, there were relatively few empirical studies examining its significance in Chinese writing development. The unique contribution of stroke order knowledge in Chinese children’s spelling development was recently reported in the study by Present Authors (2010). The present path analyses results showed that the stroke order knowledge task, which was used to assess handwriting skills in Chinese, not only had an indirect effect on text writing through spelling but had a significant direct effect on text writing. These echoed the findings by Graham et al. (1997) about the prominent role of handwriting skills in English writing. In their study, only handwriting has a significant direct effect on compositional quality, while the contribution of spelling to compositional quality was indirect through its correlation with handwriting. According to Graham et al. (1997), handwriting fluency was such a crucial mechanism skill in beginning writing because the ability to produce letter forms from memory automatically allows more attention resources for planning content, generating text and transcribing in written composition. It is quite memory-taxing to remember the strokes of each character in Chinese. As stroke order has probably evolved according to psycho-motor principles (Hoosain, 1991), it is possible that memory of stroke order helps reduce the attention resources for producing Chinese characters which in turn allow more cognitive resources for planning, translating, reviewing, and revising processes of composition.

### The role of syntactic skills in Chinese text writing

Syntactic skills were not commonly examined alongside with transcription skills and ideation in literature on writing development among early elementary grades students (e.g., Juel et al., 1986, Berninger et al., 1992) as it was hypothesized that syntactic skills, a higher level linguistic skills, were only important to writing development in intermediate grades (Berninger et al., 1991). However, results in the present study suggest that syntactic skills might play an important role in writing development even among early elementary grades students. Path analyses results showed that grade 1 syntactic skills had unique contribution to text writing in early elementary grades even after controlling for autoregressor effects and other cognitive-linguistic skills. The significance of syntactic skills in spelling development in alphabetic languages was well documented (e.g., Plaza, 2001; Plaza & Cohen, 2003; Tunmer & Hoover, 1992). Syntactic skills can enhance children’s ability to use context to identify unfamiliar words and exception words when they are practising their less than perfect grapheme-phoneme correspondences which in turn may facilitate their spelling performance (Gough & Hillinger, 1980; Jorm & Share, 1983; Tunmer, Herriman, & Nesdale, 1988). Comparatively, there were fewer attempts to explore the role of syntactic skills in text writing. Results in the present study might find support from Bishop and Snowling’s (2004) “triangle model extended” which highlighted syntactic skills as one of the two major components, the other being discourse skills, in text level language processing. Incorporating syntactic skills in models of writing seems to a promising direction for future studies on writing development.

Similar to the literature on alphabetic languages, there were few studies examining the relationships between syntactic skills and Chinese writing. Yet, syntactic skills were repeatedly found to be significant predictors of Chinese reading. As mentioned, word order has been considered the most important syntactic device in Chinese. So and Siegel’s (1997) study showed that children’s syntactic skills, in terms of their knowledge of word order, was a strong predictor of children’s performance in word recognition at grades 1–4. Recent studies also showed that word order knowledge was a significant predictor of text reading among elementary grade students in Mainland China and Hong Kong (Chik et al., 2011; Gong & Peng, 2005; Yeung et al., 2011b). Results in the present study suggest that word order knowledge, a kind of syntactic skills, is not only important to Chinese reading but also to Chinese writing development.

### Ideation and text writing

The models of text writing in the present study showed that ideation only had significant direct effect on text writing in grade 2 while syntactic skills and transcription skills had significant direct effects across grade 1 to grade 4. This pattern of results was not consistent with previous findings in the literature on alphabetic languages (e.g., Juel et al., 1986) which supported the claim that text writing in earlier grades is constrained by lower level skills (e.g., transcription skills) and higher-level linguistic skills (e.g., ideation) are more influential in higher grades (Berninger et al., 1991). One possible reason for this was that, given the complexity of the Chinese writing system, transcription skills are likely to influence text writing over a more extensive period in contrast to more transparent orthographies. Besides, there was evidence showing that the discrepancy between oral language and written language weakens the relationship between oral language skills and literacy skills (Charity, Scarborough, & Griffin, 2004; Craig, Connor, & Washington, 2003; Patton Terry, Connor, Thomas-Tate, & Love, 2010; Patton Terry & Scarborough, 2011). Cantonese, the Chinese dialect spoken by the majority of Chinese in Hong Kong, differs in significant ways from Modern Standardized written Chinese in both vocabulary and syntax. Therefore, oral language skills are expected to play a less significant role in literacy development among Chinese students in Hong Kong. Findings from two recent studies on Chinese text reading supported this claim (Yeung et al., 2011a, b). One plausible implication of these findings was that a universal model of writing development needs to take into account of the discrepancy between oral language and written language, in addition to the orthographic depth of the writing system.

### Relationship between spelling and text writing

One interesting finding is about the relationship between spelling and text writing among these Chinese children in early elementary grades. According to the simple view of writing (Juel et al., 1986; Berninger, 1996; Berninger et al., 1997, 2002b), spelling is thought to have influence on text writing. It was suggested that writing in early grades primarily consists of learning to spell and the transition of learning to write to writing to learn only appears in fourth grade. Yet, the path analyses results showed that the model (Model 2) in which the strength of the paths from spelling in a particular grade to text writing in the subsequent grade was postulated to be equal to that from text writing in a particular grade to spelling in the subsequent grade fit the data just as well as that in which no such constrains were imposed (Model 1). Our results seem to suggest that the influence of spelling and text writing is bidirectional. On one hand, spelling proficiency contributes to text writing by allowing more cognitive resources available for higher-order cognitive processes such as organization and reflection. On the other hand, text writing may facilitate spelling by consolidating the orthographic representations of the Chinese characters in two ways. First, it provides the opportunity for practicing the motoric programme involved in writing Chinese characters, which is important for lexical representation consolidation (Tan et al., 2005). Second, it allows more exposure to the sentence context in which the characters appear. According to the self learning hypothesis (Share, 1995, 1999, 2004), each successful decoding of a new word in independent reading of text is proposed to present an opportunity to acquire word-specific orthographic representations necessary for skilled visual word recognition. Knowledge of grapheme-phoneme conversion rules and sensitivity to the grammar of the language and the contexts in which specific sentences occur help the establishment of new orthographic representation. Since the phonological recoding strategy is not available to Chinese readers, the use of sentence context is expected to be important to the formation of the orthographic representations of the Chinese characters.

### Conclusion

The present study attempted to address the paucity of literature on the skills important to Chinese writing. Findings suggest that the writing development among Chinese children can be understood in light of the simple view of writing (Berninger, 2000; Berninger & Graham, 1998; Juel et al., 1986) and the “triangle model extended” (Bishop & Snowling (2004) developed for alphabetic languages. Both transcription skills and syntactic skills are predictive of Chinese text writing longitudinally among early elementary school children even after controlling for autoregressor effects. Yet, instead of spelling being a component skill contributing to text writing, the path analyses results suggest that spelling and text writing have bidirectional influences on one another. The causal relationship between transcription skills and Chinese text writing is yet to be further explored in future studies. One possible direction is to examine the effectiveness of intervention studies with explicit instructions on handwriting skills. A number of past studies showed that handwriting instruction to first and second graders with poor handwriting skills resulted in improvement in text writing in alphabetic languages (Berninger et al., 1997; Graham, Harris, & Fink, 2000; Jones & Christensen, 1999). Results in the present study suggest that the role of transcription skills in text writing in Chinese is likely to be stronger than that in alphabetic languages in view of the complexity of the Chinese writing system. The positive impacts of transcription skills instructions on text writing, if any, are likely to be even greater in Chinese. Moreover, the significance of syntactic skills in writing development was highlighted in the results of the present study. Attempts to incorporate text-level skills (e.g., syntactic skills and discourse skills) into models of writing are thus called for. Besides, we only operationalized handwriting skills in terms of the stroke order knowledge and writing in terms of compositional quality. Future studies incorporating other measures of handwriting skills (e.g., the speed of producing stroke-patterns) and writing (e.g., compositional fluency) are needed for comprehensive understanding of the role of mechanics in Chinese writing. At the same time, more research evidence is needed to validate the proposed models.

## Appendix: Sample Chinese items with English translation

1. Orthographic knowledge: Pseudo-character meaning judgment task
  - The semantic radical in  is 衤 (which carries the meaning of “clothing”)
2. Morphological awareness: Homophone awareness task
  - The words /naam4 tsi3/ (男廁 male washroom), /naam4 dzai2 /(男仔 young boy), and /naam4 gik9/ (南極 South Pole) were presented orally in the practice trial. The first two words contained the homophones sharing the same morpheme (the meaning of male), whereas the homophone in the last word had a meaning of south. To indicate the correct answers to the trial, one would have to circle the number corresponding to the order of presentation of the correct answers, i.e., 1 and 2 for the practice trial.
3. Handwriting skills: Stroke order task
